# Integrated TCM and Western medicine rehabilitation program after breast cancer surgery: a best evidence summary

**DOI:** 10.3389/fmed.2026.1930722

**Published:** 2026-09-11

**Authors:** Xuting Guo, Qiuxiang Song, Xiaoyan Zhao, Shupei Li, Xiaohan Lian, Xiaohan Yang

**Affiliations:** 1 School of Nursing, Shanxi Medical University, Taiyuan, China; 2 Department of Breast, First Hospital of Shanxi Medical University, Taiyuan, China

**Keywords:** breast cancer, evidence summary, evidence-Based nursing, integrated traditional Chinese and Western medicine, rehabilitation nursing

## Abstract

**Objective:**

To systematically identify, critically appraise, and synthesize the best available evidence on integrated TCM and Western medicine rehabilitation program after breast cancer surgery, with the aim of providing evidence-based references to support clinical nursing practice.

**Methods:**

This study was conducted and reported in accordance with the PRISMA guidelines where applicable and the evidence summary development process proposed by the Fudan University Center for Evidence-based Nursing. Guided by the “5S” evidence pyramid model, a top-down systematic search was conducted across decision-support systems, guideline websites, professional organization websites, and major databases. The search period spanned from database inception to December 2025. Two reviewers independently performed literature screening, critical appraisal, and evidence extraction.

**Results:**

A total of 29 articles were included, comprising three clinical decisions, three guidelines, eleven expert consensuses, one evidence summary, seven systematic reviews, and four randomized controlled trials. The synthesized evidence generated seven thematic domains: comprehensive assessment, postoperative functional exercise, manual lymphatic drainage, exercise prescriptions, precautions for exercise in special circumstances, appropriate traditional Chinese medicine techniques, and medical supervision and incentive strategies during exercise, yielding 27 evidence-based recommendations.

**Conclusions:**

The 27 evidence-based recommendations across seven domains provide clinically meaningful guidance for integrated TCM and Western medicine rehabilitation program after breast cancer surgery. These recommendations offer an evidence-based framework for improving clinical practice. Healthcare professionals may apply the available evidence to clinical practice tailored to individual conditions, with the aim of improving patients’ postoperative quality of life.

**Registration:**

The study protocol was registered with the Fudan University Center for Evidence-based Nursing (http://ebn.nursing.fudan.edu.cn/home). Registration number ES20259118

**Systematic Review Registration:**

http://ebn.nursing.fudan.edu.cn/home, ES20259118.

## 1 Introduction

The latest data shows that in 2022, there were 2.3 million new cases of breast cancer worldwide, accounting for approximately 12% of all new cancer cases (1). From a traditional Chinese medicine perspective, breast cancer is also known as “Ru Yan” and is one of the major cancers that seriously threaten women's health. With the advancements in cancer treatment and early disease monitoring, breast cancer has become a solid tumor with better treatment outcomes, and the survival rate and survival time of patients have improved (2). Therefore, the focus of breast cancer patients has gradually shifted from maintaining life to improving postoperative quality of life (3). Surgical treatment occupies an important position in the treatment methods of breast cancer, as it can achieve radical cure or partial resection by removing tumor tissues. However, due to its wide surgical range and large trauma area, it is prone to causing functional impairment of the affected limb in patients, manifested as shoulder joint dysfunction, breast cancer-related lymphedema, etc (4). All of these seriously affect the daily life of patients and cause them physical and mental burdens. Therefore, strengthening the guidance of postoperative rehabilitation exercises for patients and promoting the recovery of limb function and improvement of quality of life should become the focus of attention for medical staff.

The “14th Five-Year Plan for the Development of Traditional Chinese Medicine” mentions (5) that traditional Chinese medicine, Chinese traditional sports, and modern rehabilitation technologies should be deeply integrated and coordinated for application, and a rehabilitation medical system with Chinese characteristics should be accelerated to be established and developed. Within this integrated system, Western rehabilitation techniques are precisely targeted at restoring joint range of motion, muscle strength, and physical endurance, with effects that can be objectively quantified through standardized functional assessments. Meanwhile, TCM exercises focus on the coordination of breathing, body posture, and mental focus, facilitating systemic regulation by promoting blood circulation, relieving meridian stagnation, and strengthening vital energy. These mind-body practices not only improve motor function but also address common postoperative issues such as fatigue, sleep disturbances, and emotional distress, thereby enhancing overall physical and mental well-being. Currently, many domestic and foreign guidelines have provided relevant recommendations for rehabilitation programs for breast cancer patients after surgery, but these guidelines are mostly comprehensive in content and lack specificity. To the best of our knowledge, none of them consider Chinese women's needs postoperatively in the context of TCM. Therefore, this study summarizes the best evidence on the integrated traditional Chinese and Western medicine rehabilitation programs for breast cancer patients after surgery from both domestic and international sources, providing evidence-based support for clinical practice.

## 2 Method

This study was conducted as a best evidence summary. The evidence-summary process included problem formulation, evidence retrieval, source screening, methodological quality appraisal, evidence extraction, grading and synthesis. It differs from conventional systematic reviews that primarily synthesize study-level evidence from original research, and from scoping reviews that mainly map the scope, characteristics, and gaps of an evidence field.

### 2.1 Question identification

The research question was formulated as follows: What is the best available evidence for Integrated TCM and Western medicine rehabilitation program after breast cancer surgery? A structured evidence-based question was developed according to the PIPOST framework (6). P (population): The target population of the evidence application comprised postoperative patients with breast cancer. I (Interventions): The intervention referred to integrated TCM and Western medicine rehabilitation program. P (Professionals): The applied evidence professional included breast cancer patients, caregivers, and medical staff. O (Outcome):The primary outcomes were the Postoperative Functional Exercise Compliance Scale for Breast Cancer, shoulder joint range of motion Scale, and Upper Limb Dysfunction Score Scale; the secondary outcomes were the Quality of Life Measurement Scale for Breast Cancer Patients, Cancer Fatigue Scale, and TCM Syndrome Score. S (Setting): The application setting encompassed hospitals, households and communities. T (Type of evidence): The type of evidence included expert consensus, clinical decision-making, guidelines, evidence summaries, systematic reviews, and original studies closely related to the research topic.

### 2.2 Search strategy

**Figure 1:**
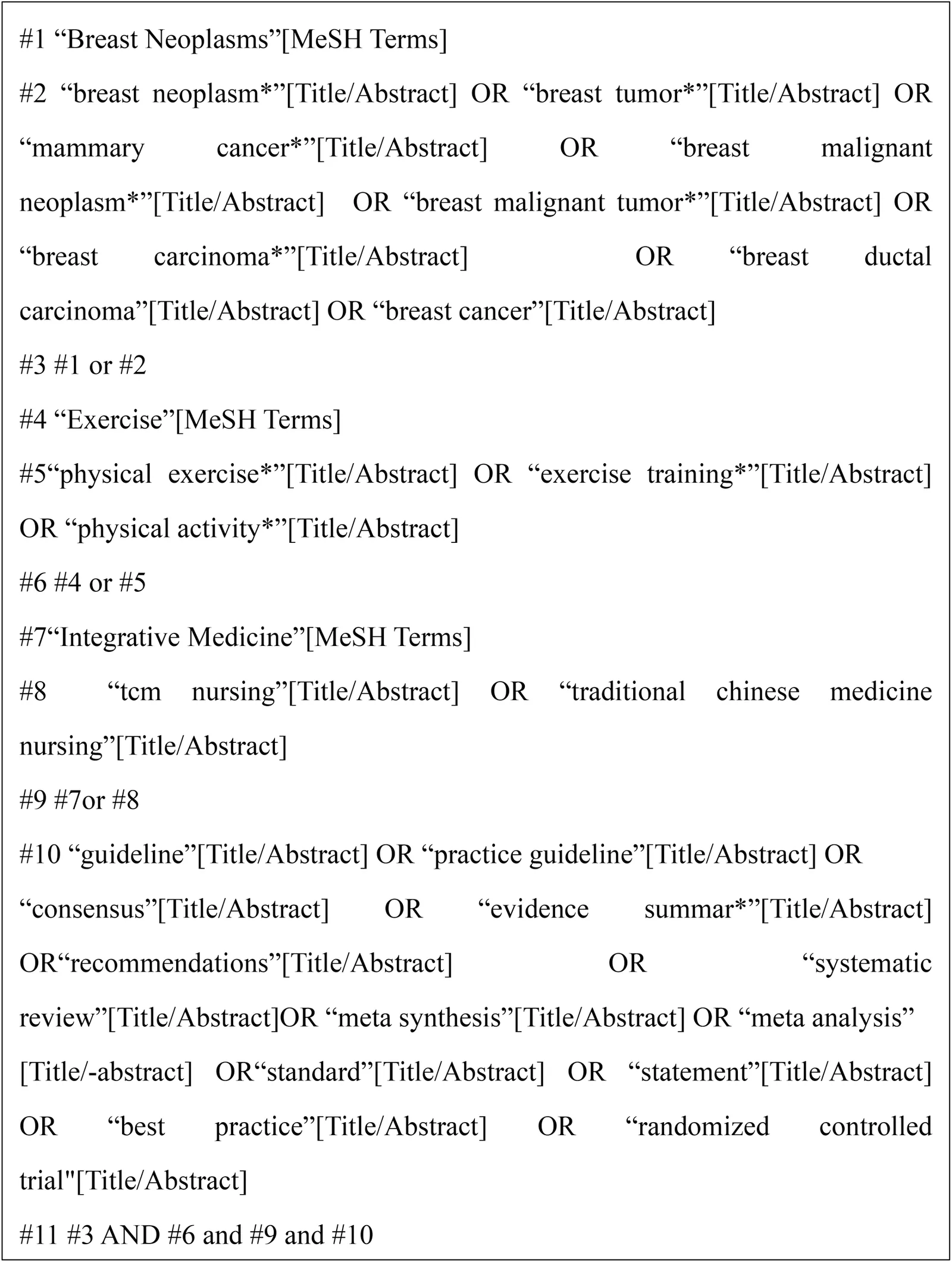
Pubmed search strategy.

According to the evidence “5S” pyramid model (7), searches were conducted following a top-down approach. The search was conducted in the following databases: BMJ Best Practice, UpToDate, World Health Organization website (WHO), Registered Nurses' Association of Ontario (RNAO), Guidelines International Network (GIN), New Zealand Guidelines Group (NZGG), American Society of Clinical Oncology (ASCO), National Institute for Health and Care Excellence (NICE), Joanna Briggs Institute (JBI) Center for Evidence-Based Health Care Database, Chinese Medicine (CACM), Medlive, PubMed, CINAHL, Cochrane Library, Embase, China National Knowledge Infrastructure (CNKI), Wanfang Data, Chinese Science and Technology Journal Database(VIP), Chinese Biology Medicine(CBM) database were searched to collect the relevant articles. The search period covered the database establishment to December 2025. The search terms included “Breast Neoplasm”, “Breast Tumor”, “Mammary Cancer”, “Breast Malignant Neoplasm”, “Breast Malignant tumor”, “Breast Carcinoma”, “ Breast Ductal Carcinoma”, “Breast Cancer”, “Exercise”, “Physical Activity”, “Exercise training”, “Physical Therapy”, “Rehabilitation Exercise”, “Resistance Training”, “Strength Training”, “TCM Nursing”, “integrative medicine”, “traditional Chinese medicine nursing”, “guideline”, “consensus”, “evidence summary”, “systematic review”, “Meta-analysis” and “randomized controlled trial”. The databases were searched using a combination of subject headings with free-text words. PubMed was used as an example of the English database, and the search strategy is shown in Figure 1.

### 2.3 Literature inclusion and exclusion criteria

Inclusion criteria were as follows: (a) the study population consisted of breast cancer patients, (b) the study contents involved functional exercises, postoperative movements, and related traditional Chinese medicine nursing techniques, (c) the types of literature are expert consensus, clinical decisions, guidelines, systematic reviews, evidence summaries, and randomized controlled trials; (d) language was either Chinese or English (Chinese was necessary for the TCM-focused evidence base, and Chinese and English were the languages in which the research team could reliably conduct full-text screening, data extraction, and critical appraisal).

Exclusion criteria were as follows: (a) the full text cannot be obtained, (b) interpretation, analysis and repeated publication of clinical guidelines, (c) insufficient information.

### 2.4 Quality assessment tools

Different instruments were selected according to the type of evidence source. Guidelines were evaluated using the Clinical Guidelines for Research and Evaluation II (AGREE II) tool (8). Evidence summaries and clinical decisions were assessed using the Critical Appraisal for Summaries of Evidence (CASE) tool (9). Expert consensus was evaluated according to JBI evidence-based health care centers (2016) (10). Systematic review was evaluated using the measurement tool to assess systematic review II (AMSTAR II) (11). Randomized controlled trials (RCTs) were appraised using the Cochrane Risk of Bias 2.0 (RoB 2.0) tool, which evaluates the randomization process, deviations from intended interventions, missing outcome data, outcome measurement, and selection of the reported result (12). The quality evaluations were independently completed by two researchers who had received training in evidence-based nursing. In case of disagreement, consensus was reached through discussion, or a third researcher participated and formed the final conclusion.

### 2.5 Evidence extraction and integration

Two researchers extracted the content of the final included literature, and the third researcher verified the semantic and content of the extracted results. If there were any misunderstandings due to translation, they were discussed by senior experts with professional English background within the team until a consensus was reached. The evidence summary follows the following principles: ① If the contents are the same, retain the concise and clear recommendations; ② If the contents are complementary, combine them based on logical relationships; ③ If the contents conflict: follow the principle of giving priority to the latest and high-quality evidence; ④ If the contents are independent of each other, fully retain the original expressions. Evidence was graded using the JBI Centre for Evidence-Based Medicine 2014 evidence grading system. Evidence levels ranged from Level 1 to Level 5, with Level 1 representing the highest level of evidence. Specifically, in this summary, Systematic reviews of RCTs were assigned Level 1a; RCTs were assigned Level 1c; guidelines and expert consensus were assigned Level 5b. Recommendations were further classified according to effectiveness, feasibility, appropriateness, and clinical significance, and were categorized as Grade A (strong recommendation) or Grade B (weak recommendation) (13).

## 3 Results

### 3.1 Literature search results and general information

**Figure 2:**
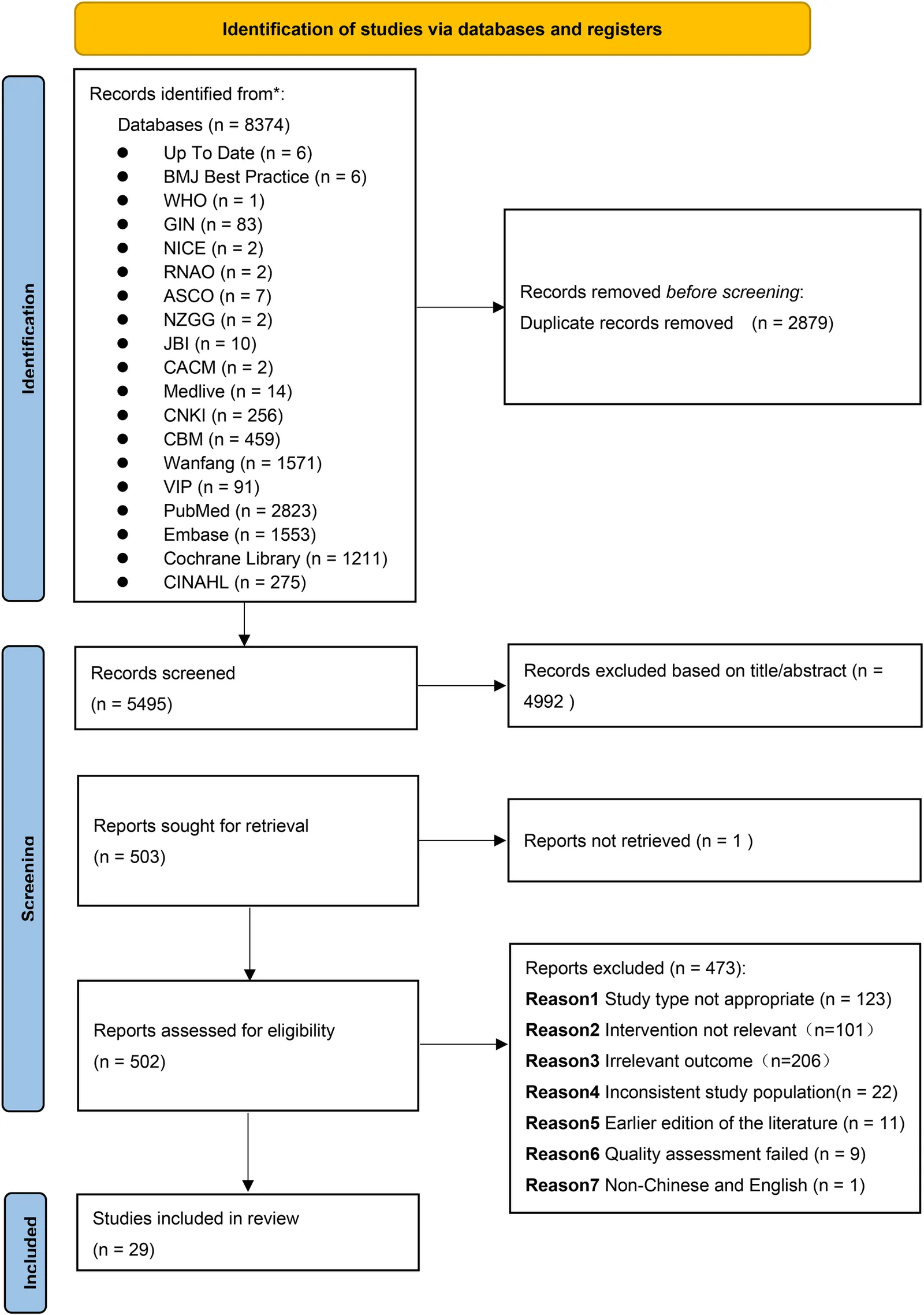
PRISMA flow diagram of literature search and selection process.

A total of 8,374 articles were ultimately included. After removing duplicates and reviewing the titles, abstracts, and full texts, 29 articles were ultimated included. These comprised three clinical decisions (15–17), three guidelines (20, 24, 38), eleven expert consensus (14, 18, 19, 21–23, 26–29, 37), one evidence summary, (42) seven systematic reviews (25, 30–33, 39, 40), and four randomized controlled trials (34–36, 41). A literature screening flowchart is shown in Figure 2, and general characteristics of the included literature are presented in Table 1.

**Table 1:** Characteristic of included literature (*n* = 29).

Included literature	Publication year	Literature source	Literature type	Literature topic
Brunelle et al. (15)	2025	UpToDate	Clinical decision	Screening and prevention of BCRL
Ligibel et al. (16)	2025	UpToDate	Clinical decision	Effects of physical activity, diet and body weight on cancer patients
Greenlee et al. (17)	2025	UpToDate	Clinical decision	Complementary, alternative and integrative treatments for tumors, as well as potential risks and hazards
Lu et al. (38)	2025	PubMed	Guideline	Comprehensive therapy improves the reported outcomes of breast cancer patients
The Breast Cancer Professional Committee of the Chinese Cancer Society (24)	2023	Medlive	Guideline	Diagnosis and Treatment of Breast Cancer
National Tumor Quality Control Center Breast Cancer Expert Committee (20)	2022	Medlive	Guideline	Breast Cancer Follow-up and Management for Health Care
Health China Research Center Cancer Prevention and Control Expert Committee (18)	2025	Medlive	Expert consensus	Comprehensive and specialized integrated management of concurrent diseases associated with breast cancer
Chinese expert group specializing in oncology (14)	2025	Medlive	Expert consensus	Integrated Traditional Chinese and Western Medicine Rehabilitation for Breast Cancer in Community Hospitals
Chinese expert group specializing in oncology (22)	2025	Medlive	Expert consensus	Tumor rehabilitation
The Breast Cancer Integrated Nursing Professional Committee of the Chinese Anti-Cancer Association (27)	2025	Medlive	Expert consensus	Body management for breast cancer patients
The Breast Cancer Integrated Nursing Professional Committee of the Chinese Anti-Cancer Association (28)	2025	Medlive	Expert consensus	Home-based exercises for BCRL prevention and treatment
Shanghai Cancer Association (21)	2024	Medlive	Expert consensus	Tumor Integrated Rehabilitation Management
Beijing Society for Breast Disease Prevention and Treatment - Professional Committee of Integrated Traditional Chinese and Western Medicine (19)	2023	Medlive	Expert consensus	Combined traditional Chinese and Western medicine-assisted intensive treatment for triple-negative breast cancer
Chinese Rehabilitation Medical Association (23)	2023	Medlive	Expert consensus	Exercise rehabilitation for cancer patients in China centered on functional impairment
The Professional Committee of Oncology Nutrition and Support Therapy of the Chinese Anti-Cancer Association (26)	2022	Medlive	Expert consensus	Exercise Therapy for Malignant Tumor Patients in China
The Project Team for Formulating and Implementing Diagnosis and Treatment Plans and Clinical Paths for Key Diseases in Traditional Chinese Medicine (29)	2022	CNKI	Expert consensus	Traditional Chinese Medicine Clinical Pathway and the Diagnosis and TreatmentScheme of Ruyan
The Oncology Committee of the Chinese Association of Integrated Traditional Chinese and Western Medicine (37)	2021	Medlive	Expert consensus	Integrated Traditional Chinese and Western Medicine Treatment for Breast Cancer
Wei et al. (42)	2022	CNKI	Evidence summary	Self-management of BCRL
Guo et al. (25)	2024	CNKI	Systematic review	Effects of different exercise therapies on breast cancer patients
Wu et al. (30)	2023	CNKI	Systematic review	The therapeutic effect of virtual reality technology on the rehabilitation of breast cancer patients
Yang et al. (31)	2019	CNKI	Systematic review	Manual lymphatic drainage for the prevention and treatment of BCRL
Li et al. (32)	2023	CNKI	Systematic review	Effects of virtual reality technology on the intervention of breast cancer patients
Zhao et al. (33)	2023	Wanfang	Systematic review	The impact of resistance training on breast cancer patients
Zhang et al. (39)	2025	PubMed	Systematic review	Digital behavior change intervention promotes exercise among breast cancer patients
Blount et al. (40)	2021	PubMed	Systematic review	The impact of wearable technology on the rehabilitation of breast cancer patients
Cao et al. (34)	2021	Wanfang	Randomized controlled trials	The impact of progressive comprehensive rehabilitation on breast cancer patients
Sun et al. (35)	2015	CNKI	Randomized controlled trials	Effects of moxibustion on the functional rehabilitation of the upper limbs after breast cancer surgery
Xu et al. (36)	2021	CNKI	Randomized controlled trials	Effects of Eight Posture Exercises for Shoulder Joints on the Postoperative Rehabilitation of Breast Cancer Patients
Xu et al. (41)	2019	CNKI	Randomized controlled trials	Effects of Five Elements Music Therapy for cancer patients

### 3.2 Quality assessment results of the included literature

#### 3.2.1 Quality assessment result of guidelines

This study included three clinical practice guidelines and the quality evaluation results are shown in Table 2.

**Table 2:** Methodological quality evaluation of the guidelines (*n* = 3).

Inculusion guidelines	Normalized percentage of scores (%)						≥60%	≥30%	Recommendation level
	Scope and Purpose	Involved personnel	Preciseness of guideline development	Clarity of presentation	Applicability	Independence of writing			
Lu et al (38)	97.22	100	92.71	100	92.71	100	6	6	A
The Breast Cancer Professional Committee of the Chinese Cancer Society (24)	91.67	72.22	35.42	97.22	70.83	91.67	5	6	B
National Tumor Quality Control Center Breast Cancer Expert Committee (20)	100	66.67	22.92	94.44	43.75	91.67	4	5	B

#### 3.2.2 Quality assessment result of the evidence summary and clinical decision

This study included a total of 1 evidence summary and 3 clinical decision. The quality assessment results are presented in Table 3.

**Table 3:** Methodological quality evaluation of evidence summary and clinical decision (*n* = 4).

Evidence summary and clinical decision	Evaluation entry									
	①	②	③	④	⑤	⑥	⑦	⑧	⑨	⑩
Brunelle et al (15)	Yes	Yes	Yes	No	Yes	Yes	Yes	Yes	Yes	Yes
Ligibel et al (16)	Yes	Yes	Yes	No	Yes	Yes	Yes	Yes	Yes	Yes
Greenlee et al (17)	Yes	Yes	Yes	No	Yes	Yes	Yes	Yes	Yes	Yes
Wei et al (42)	Yes	Yes	Yes	Yes	Yes	Yes	Yes	Yes	Yes	Yes

(1) Is the scope and application of the abstract specific? (2) Is the identity of the author of this abstract transparent? (3) Is the reviewer of the abstract transparent? (4) Is the search method transparent and comprehensive? (5) Is the evidence grading system transparent and translatable? (6) Are these suggestions clear? (7) Have these suggestions been appropriately cited? (8) Are the suggestions up-to-date? (9) Is this summary fair? (10) Can this summary be applied to your patients?.

#### 3.2.3 Quality assessment result of expert consensuses

This study included a total of 11 expert consensuses. The quality assessment results are presented in Table 4.

**Table 4:** Methodological quality evaluation of expert consensus (*n* = 11).

Expert consensus	Evaluation entry					
	①	②	③	④	⑤	⑥
Health China Research Center Cancer Prevention and Control Expert Committee (18)	Yes	Yes	Yes	Yes	Yes	Unclear
Chinese expert group specializing in oncology (14)	No	Yes	Yes	Yes	Yes	Unclear
Chinese expert group specializing in oncology (22)	Yes	Yes	Yes	Yes	Yes	Unclear
The Breast Cancer Integrated Nursing Professional Committee of the Chinese Anti-Cancer Association (27)	Yes	Yes	Yes	Yes	Yes	Unclear
The Breast Cancer Integrated Nursing Professional Committee of the Chinese Anti-Cancer Association (28)	Yes	Yes	Yes	Yes	Yes	Unclear
Shanghai Cancer Association (21)	Yes	Yes	Yes	Yes	Yes	No
Beijing Society for Breast Disease Prevention and Treatment - Professional Committee of Integrated Traditional Chinese and Western Medicine (19)	Yes	Yes	Yes	Yes	Yes	No
Chinese Rehabilitation Medical Association (23)	Yes	Yes	Yes	Yes	Yes	No
The Professional Committee of Oncology Nutrition and Support Therapy of the Chinese Anti-Cancer Association (26)	Yes	Yes	Yes	Yes	Yes	Unclear
The Project Team for Formulating and Implementing Diagnosis and Treatment Plans and Clinical Paths for Key Diseases in Traditional Chinese Medicine (29)	Yes	Yes	Yes	Yes	Yes	No
The Oncology Committee of the Chinese Association of Integrated Traditional Chinese and Western Medicine (37)	Yes	Yes	Yes	Yes	Yes	No

(1) Has the source of the viewpoint been clearly indicated? (2) Does the viewpoint originate from influential experts in the field? (3) Does the proposed viewpoint focus on the interests of the relevant research subjects? (4) Is the stated conclusion based on the analysis results? Is the expression of the viewpoint logical? (5) Has it referred to other existing literature? (6) Are there any inconsistencies between the proposed viewpoint and previous literature?.

#### 3.2.4 Quality assessment result of systematic reviews

This study included a total of 7 systematic reviews. The quality assessment results are presented in Table 5.

**Table 5:** Methodological quality evaluation of systematic review (*n* = 7).

Systematic review	Evaluation entry															
	①	②	③	④	⑤	⑥	⑦	⑧	⑨	⑩	⑪	⑫	⑬	⑭	⑮	⑯
Guo et al. (25)	Yes	No	Yes	Yes	Yes	Yes	Yes	Yes	Yes	No	Yes	Yes	Yes	Yes	Yes	No
Wu et al. (30)	Yes	No	Yes	Yes	Yes	Yes	Yes	Yes	Yes	No	Yes	Yes	Yes	Yes	Yes	No
Yang et al. (31)	Yes	No	Yes	Yes	Yes	Yes	Yes	Yes	Yes	No	Yes	Yes	Yes	Yes	Yes	No
Li et al. (32)	Yes	No	Yes	Yes	Yes	Yes	No	Yes	Yes	No	Yes	Yes	Yes	Yes	Yes	No
Zhao et al. (33)	Yes	No	Yes	Yes	Yes	Yes	Yes	Yes	Yes	No	Yes	Yes	Yes	Yes	Yes	Yes
Zhang et al. (39)	Yes	No	Yes	Yes	Yes	Yes	Yes	Yes	Yes	No	Yes	Yes	Yes	Yes	Yes	No
Blount et al. (40)	Yes	No	Yes	Yes	Yes	Yes	Yes	Yes	Yes	No	Yes	Yes	Yes	Yes	Yes	Yes

(1) Do the research questions and inclusion criteria include the PICO components? (2) Has it been stated that the research methods for the systematic review were determined before its implementation, and if there are any inconsistencies with the research plan, have they been explained? (3) When including the literature, has the type of studies included been stated? (4) Has a comprehensive search strategy been adopted? (5) Has double-blind literature selection been used? (6) Has double-blind data extraction been used? (7) Has an exclusion list of literature been provided and the reasons explained? (8) Has the included studies been described in detail? (9) Has an appropriate tool been used to assess the risk of bias for each included study? (10) Has the funding source for each included study been reported? (11) Has appropriate statistical methods been used to combine the research results? (12) Has the potential impact of the risk of bias in the included studies on the results of the Meta-analysis and other evidence synthesis results been evaluated? (13) When interpreting or discussing each research result, has the risk of bias in the included studies been considered? (14) Has any heterogeneity in the research results been reasonably explained and discussed? (15) Has the quantitative combination fully investigated the publication bias and discussed its possible impact on the results? (16) Has all potential sources of conflicts of interest been reported?.

#### 3.2.5 Quality assessment results of randomized controlled trials

This study included a total of 4 randomized controlled trials. The overall risk-of-bias judgments and domain-specific assessment results for each trial are presented in Table 6.

**Table 6:** Methodological quality evaluation of randomized controlled trials (*n* = 4).

Randomized controlled trials	Evaluation entry				
	Randomization process	Deviations from the intended interventions	Missing outcome data	Measurement of the outcome	Selection of the reported result
Cao et al. (34)	Some concerns	Some concerns	Some concerns	Low risk	Low risk
Sun et al. (35)	Low risk	Some concerns	Some concerns	Low risk	Low risk
Xu et al. (36)	High risk	Some concerns	Some concerns	Low risk	Low risk
Xu et al. (41)	Low risk	Some concerns	Some concerns	Low risk	Low risk

### 3.3 Evidence summary results

This study encompasses comprehensive assessment, functional exercise of the affected limb, manual lymphatic drainage, exercise prescriptions, precautions for special circumstances during exercise, appropriate traditional Chinese medicine techniques, medical supervision and incentive strategies during exercise. In total, 27 pieces of evidence are presented in Table 7.

**Table 7:** Evidence summary for integrated traditional Chinese and western medicine exercise rehabilitation after breast cancer surgery.

Category	Description of evidence	Evidence level	Grade of recommendation
Comprehensive assessment	1. Form a multidisciplinary team consisting of doctors from the rehabilitation department, oncology department, and traditional Chinese medicine department, as well as rehabilitation therapists, psychological counselors, and specialized nurses, etc. (14, 18, 22)	5b	A
	2. Motor ability assessment:(1) Aerobic motor ability assessment: 6-minute walk test, Borg score, pulmonary and cardiac function evaluation grade;(2) Physical ability assessment: Simple physical function assessment scale;(3) Upper limb function assessment: DASH simplified scoring table, upper arm circumference measurement, shoulder joint range of motion, muscle strength examination (including muscle strength and muscle tone examination);(4) For patients with bone metastasis or potential risk of osteoporosis, the bone health status assessment should be conducted by a sports medicine professional. (14, 23)	5b	A
	3. Risk classification for exercise:(1) Low risk: Patients with early-stage tumors without comorbidities, conducting rehabilitation exercises in the community or at home;(2) Moderate risk: Patients with peripheral neuropathy, muscle and bone disorders, lymphedema, etc., engaging in moderate-intensity exercises under medical supervision;(3) High risk: For patients with heart and lung diseases, unhealed thoracic and abdominal surgeries, severe malnutrition, and deteriorating physical condition, a multi-disciplinary team must develop an exercise prescription, and the exercise must be carried out under medical supervision. (21)	5b	A
Postoperative functional exercise	4. Start time: Generally, it is 24 h after the surgery. The first session should last for 10–15 min. From then on, each session should be 15–30 min long. The exercise should be carried out under the guidance of a professional therapist. After discharge, the patient can perform it independently. (34)	1c	A
	5. Progressive principle: Start performing exercises such as clenching fists, extending fingers, and flexing the wrist joint 1–2 days after the surgery; carry out active forearm extension and flexion movements 3–4 days after the surgery; practice touching the opposite shoulder and the same ear with the affected hand 5–7 days after the surgery; carry out activities of raising, extending, and flexing the shoulder joint 8–10 days after the surgery; after 10 days, gradually start performing wall climbing exercises for the shoulder joint and using assistive devices for training. (24)	5b	A
	6. Standard requirements: One month after the incision heals, the affected upper limb should be able to extend, lift up, and reach over the head to touch the ear on the opposite side; within 1–2 months, the shoulder joint function of the affected side should recover to the pre-operative level or be similar to that of the healthy side. After reaching the standard, rehabilitation training should still be carried out consistently. (24)	5b	A
	7. Notes: Within one week after the surgery (especially before the removal of the axillary drainage tube), strictly limit the abduction movement of the shoulder joint; if there is subcutaneous effusion or the drainage tube remains in place for more than one week, appropriately reduce the frequency of functional exercises and limit the range of shoulder joint movement. For patients who have undergone skin grafting, expanders, prosthesis implantation, or autologous myocutaneous flap breast reconstruction surgery, in the early postoperative period, avoid large abduction and elevation movements. (24)	5b	A
Manual lymphatic drainage	8. Early manual lymphatic drainage for patients can promote the return of lymph fluid and prevent the occurrence of lymphedema. It is recommended to perform a self-manual lymphatic drainage once after exercise. The specific massage sequence is as follows: supraclavicular fossa - bilateral neck - healthy side axilla, chest - affected side chest, shoulder, upper arm, forearm, wrist joint, fingers. When massaging, be sure not to apply excessive force. (14, 28, 31)	1a	A
Exercise prescriptions	9. Professional staff formulate individualized exercise prescriptions based on the patient’s condition. The prescriptions are designed using the elements of frequency, intensity, time and type (FITT) to determine the exercise program. (21, 26)	5b	A
	10. Avoid sitting still. (16, 20, 24)	5b	A
	11. Aerobic exercise: It is recommended that patients engage in physical activity for 3 to 5 days per week, accumulating a total of 150 min of moderate-intensity or 75 min of high-intensity aerobic exercise. The intensity of the exercise is monitored through heart rate or subjective exertion level (RPE): moderate-intensity exercise should be controlled at 65% - 75% of the maximum heart rate, or RPE maintained at 12–13 points; high-intensity exercise should be controlled at 76% - 95% of the maximum heart rate, or RPE maintained at 14–17 points; or patients can be instructed to judge the intensity of the exercise based on their own perceived level of exertion: during moderate-intensity exercise, one can speak but not sing; during high-intensity exercise, one cannot speak complete sentences. (20, 23, 24, 26, 38)	5b	B
	12. Resistance training: At least 2 times per week. The resistance intensity should be set at 60% - 80% of the 1 repetition maximum (1-RM) of a single attempt. The load can be gradually increased based on the patient’s actual tolerance. Each training session should cover all major muscle groups of the body, such as the chest, shoulders, back, waist, abdomen, hips, and lower limbs. The interval between two training sessions for the same muscle group should not be less than 48 h. The rest time between sets should be no less than 60 s. The duration of each training session should be controlled within 10–15 min. (23, 33, 38)	1a	A
	13. Flexibility exercises: Static stretching is recommended. During training, try to cover all major muscle groups of the body. Each stretching movement should be repeated 2–4 times, with the duration within 60 s. It is suggested to perform 2–3 times per week. If conditions permit, regular practice can be done daily. (23, 26)	5b	A
	14. Traditional mind-body exercises such as Tai Chi, Baduanjin, Qigong and Yoga can effectively alleviate physical discomfort and related psychological symptoms in patients. (23, 24)	5b	B
	15. It is recommended that patients engage in low to moderate-intensity exercise in the morning and evening, and moderate to high-intensity exercise in the afternoon. The total duration of exercise should be 30 to 90 min per day. The duration of exercise should be gradually increased according to the body’s adaptation. (27)	5b	A
Precautions for exercise in special circumstances	16. For patients with osteoporosis, bone metastasis or those who have previously received hormone therapy, a comprehensive clinical assessment and medication treatment must be completed before exercising. During resistance training, direct weight-bearing on the affected area should be avoided; high-impact training and movements such as excessive flexion, extension, and rotation of the trunk should be prohibited; close monitoring of pain changes at the affected site should be conducted before, during, and after the exercise. (23–26)	5b	B
	17. Patients with combined lymphedema should undergo training under the supervision of professionals, gradually increasing the movement volume and intensity of the affected limb. At the same time, close monitoring of the limb circumference changes is necessary. It is recommended to wear pressure sleeves during exercise. (15, 26, 28, 42)	1a	A
	18. Patients undergoing radiotherapy should focus on evaluating the range of motion of the joints around the radiotherapy area and prioritize local flexibility training; avoid exposure to chlorine-containing environments, such as participating in swimming pool activities; closely monitor the changes in the skin and soft tissues of the radiotherapy area before and after training. (23)	5b	B
	19. For patients with indwelling catheters in their bodies, resistance exercises should be avoided in the muscle groups where the catheter is located, and vigorous movements of the limb on the side where the catheter is placed should also be prevented. For non-implanted indwelling catheters, swimming and other activities that may cause cross-infection should also be prohibited. (23–26)	5b	B
Appropriate traditional Chinese medicine techniques	20. Acupoint massage: Using thumb rubbing or elbow rubbing techniques, operate along the regions where the hand’s three yin meridians and three yang meridians run from distal to proximal. At the same time, apply point stimulation to acupoints such as Jianyu, Tianzong, Shangjiao, Shangzheng, Quanchi, Wanguan, Neiguan, Shaohuan, and Xiaohai. Each acupoint should be massaged for 3–5 min, with a frequency of 30 times per minute. It is recommended to perform this once in the morning and once in the evening. (29)	5b	B
	21. Moxibustion: The selected acupoints on the affected upper limb include Jianjing, Jianliao, Jianzhen, Binao, and Ashi points. The ignited moxa stick is held 2–3 cm above the skin surface for smoking and heating. After a warm sensation is perceived, the moxa stick can be moved evenly in a front-back and left-right direction or rotated, until the local skin shows a mild flushing reaction. Each acupoint is treated for 3–5 min, with a total session duration of 15–20 min. The treatment is administered twice daily (once in the morning and once in the afternoon) for a continuous course of 14 days. (35)	1c	B
	22. Auricular Seed Therapy: The auricular acupoints selected include Liver, Spleen, Heart, and Sanjiaoto harmonize the zang-fu viscera, soothe the liver and regulate qi, and nourish and regulate qi and blood. Additionally, the acupoints Sympathetic, Shenmen, Occiput, Endocrine, Subcortex, and Neurasthenic Point are chosen to calm the mind and regulate qi, as well as to tranquilize the mind and relieve anxiety. After precise localization, the auricular plasters carrying Wangbuliuxing seeds are firmly affixed to the corresponding acupoints. The seeds are then pressed with the opposing surfaces of the index fingers and thumbs, applying moderate pressure until a local sensation of soreness, numbness, distension, or pain is elicited. (37)	1c	B
	23. Acupuncture: Each session lasts 20–30 min, administered 1–2 times per week. Six weeks constitute one course of treatment, and the therapy may be continued for 1–3 consecutive courses. Acupoint selection is determined according to different syndrome patterns:(1) Spleen-Stomach Disharmony Pattern: Zhongwan, Jianli, Liangmen, Tianshu, and Zusanli;(2) Qi-Blood Deficiency Pattern: Qihai, Pishu, Weishu, and Zusanli;(3) Qi-Yin Deficiency Pattern: Zusanli, Pishu, Weishu, and Taixi;(4) Chong-Ren Disregulation Pattern: Guanyuan and Sanyinjiao (SP6);(5) Liver-Kidney Deficiency Pattern: Taixi, Shenshu, and Ganshu. (14, 38)	1a	B
	24. Traditional Chinese Exercise Therapies: Tai Chi (Banlan Chui, Zuo Lan Que Wei, You Lan Que Wei, Zuo Xia Shi Du Li, You Xia Shi Du Li, Yun Shou), Ba Duan Jin (Cuan Quan Nu Mu Zeng Qi Li, Tiao Li Wei Chang Xu Dan Ju, Shuang Shou Pan Zu Gu Shen Yao, Zuo You Kai Gong Si She Diao), Wuqinxi (Niao Fei, Xiong Huang, Lu Ben, Hu Pu). These shoulder movements can effectively enhance upper limb motor function in patients. (19, 25, 36, 38)	1a	A
	25. Five-element Music Therapy: Based on the “Guidelines for Traditional Chinese Medicine Diagnosis and Treatment of Malignant Tumors”, the patient’s condition is diagnosed using traditional Chinese medicine theory. The music prescription is formulated by combining the five elements, five emotions, five tones, and the corresponding relationships between the five zang-organs. The Traditional Chinese Music Box Set issued by the Chinese Medical Association Audio-Visual Publishing House is selected as the audio source. The patient is instructed to coordinate breathing regulation, muscle relaxation, and guided music imagination. The volume is set at 30–40 decibels, 30 min per session, twice a day. (17, 41)	1c	A
Medical supervision and incentive strategies during exercise	26. Medical staff should enhance the education on sports safety for patients and their families, and provide continuous supervision, guidance and regular follow-ups during the sports implementation process. It is recommended that patients consult with a specialist physician or a professional sports instructor every 3–6 months to conduct a comprehensive assessment of their current physical activity and formulate targeted improvement plans accordingly. (20, 28)	1a	A
	27. Introduce digital behavioral change interventions (such as virtual reality technology, wearable devices, pedometers, etc.) as supplementary means for rehabilitation exercises. (30, 32, 39, 40)	1a	A

## 4 Discussion

### 4.1 Precise assessment is the key to enhancing the scientific nature of rehabilitation exercises

Accurate and comprehensive assessment directly influences the formulation of individualized exercise plans and should be integrated throughout the entire rehabilitation exercise process, mainly including team building, exercise ability assessment, and exercise risk classification. Evidence 1 suggests establishing a multidisciplinary team, with oncologists conducting the initial assessment and risk stratification. If adverse reactions are detected during diagnosis and treatment, they should be promptly referred to exercise medicine professionals and other necessary supporting disciplines to prevent the occurrence of adverse events (14). Evidence 2 systematically summarizes specific assessment tools related to exercise ability, such as aerobic exercise, physical function, and upper limb function, but most of them focus on assessing a particular aspect, such as balance ability, muscle strength, and cardiopulmonary endurance, which are difficult to comprehensively reflect the patient's exercise functional status. Moreover, there is currently a lack of high-quality clinical research to directly verify the quantitative correspondence between “assessment—exercise prescription”, that is, which assessment results correspond to which exercise frequency, intensity, duration, and type. This issue of the gap between assessment and prescription is precisely the current clinical problem that urgently needs to be solved. Subsequently, such research should be actively carried out to provide evidence-based support for postoperative rehabilitation exercise. In addition to exercise ability assessment, exercise self-efficacy is also a key indicator for evaluating the patient's exercise level, directly related to the patient's exercise compliance and planning ability (43). Therefore, we should select scientific and appropriate assessment tools to accurately grasp the patient's exercise self-efficacy. Rogers et al. (44) developed a specific exercise self-efficacy scale for breast cancer patients in 2006, but this scale has not yet been introduced in China, and its applicability in the Chinese population still needs to be verified. In the future, it can be combined with the cultural background and clinical reality of China to develop a standardized and localized breast cancer patient-specific exercise self-efficacy evaluation tool with local characteristics. Evidence 3 further classifies patients' exercise risks into low, medium, and high levels, providing a basis for formulating individualized exercise plans. Therefore, before the emergence of high-quality evidence, it is recommended to rely on the multidisciplinary team to base the risk classification on exercise ability assessment, combined with tumor diagnosis and treatment conditions, exercise self-efficacy, etc., and implement personalized exercise adjustments based on the patient's own exercise habits. At the same time, digital means such as wearable devices can be used to achieve dynamic assessment and real-time feedback, compensating for the limitations of static assessment.

### 4.2 The combination of traditional Chinese and western medicine is the key to formulating individualized exercise plans

Evidence 4–15 summarizes modern rehabilitation techniques, including aerobic exercise, resistance exercise, shoulder-hand-elbow movement, manual lymphatic drainage, etc., but it only focuses on the training of muscle strength and joint range of motion, and its role in regulating the internal environment of the human body is not obvious. Evidence 20–25 summarizes traditional Chinese medicine techniques, including acupoint massage, moxibustion, ear bean pressing, traditional Chinese medicine exercises, and five-element music therapy. From the perspectives of breathing, body adjustment, and mind regulation, it can achieve the goal of treating both the symptoms and the root causes and maintaining the body's form and spirit. The combination of traditional Chinese and Western medicine reflects an overall view that emphasizes both functional rehabilitation and emotional regulation. Liu et al. (45) combined breathing exercises with acupoint massage and applied it to elderly patients with pneumoconiosis. The results showed that it could effectively improve their pulmonary ventilation function and exercise ability. Yang et al. (46) combined traditional exercises such as the Eight Pieces of Strength with modern sports to create the Five-Body Balance Exercise, which covers comprehensive training of consciousness, breathing, and the body. The results showed that compared with conventional aerobic exercise, this exercise has higher safety and can better improve the physiological indicators and quality of life of patients with obesity-related hypertension. Xiang et al. (47) combined sensory integration training in modern rehabilitation treatment with the five-element music therapy in traditional Chinese medicine treatment to intervene in children with comprehensive developmental delay. The results showed that this combined scheme could effectively promote the improvement of the children's neuro-psychological development level. Professor Zhu (48) advocated the “312 Meridian Exercise Method”, which is a self-medical care method integrating traditional Chinese meridian theory and modern sports physiology. It has been widely applied in chronic disease management and has positive significance in objective indicators of chronic diseases and improvement of quality of life. Jin et al. (49) combined AR sensory movement with acupoint massage, which can effectively alleviate fatigue and other symptoms of breast cancer patients and greatly improve the compliance of patients with rehabilitation. Although the combination of traditional Chinese and Western medicine has achieved many positive results in the field of rehabilitation exercise, in clinical practice, there is still a pain point of “surface integration": some combination schemes lack unified efficacy standards and are difficult to form a scalable standard (50); even there is a formal problem of “merely combining for the sake of combining”, failing to truly leverage the advantages of both in addressing the pain points of disease diagnosis and treatment. The combination of traditional Chinese and Western medicine is not a mechanical assembly, but an organic system of complementary advantages and synergistic enhancement. Future research should conduct more high-quality randomized controlled trials to explore the integration path of the overall view of traditional Chinese medicine and modern rehabilitation objective indicators, establish a standardized efficacy evaluation system, and promote the transition from “formal integration” to “essential integration” of traditional Chinese and Western medicine combination.

### 4.3 Strengthening process supervision is a powerful guarantee for improving rehabilitation efficiency

The patient's exercise compliance and regularity are important influencing factors determining the rehabilitation outcome. Studies have shown that the majority of breast cancer patients fail to meet the exercise standards recommended by the World Health Organization (WHO) (51). Murray et al. (52) found that post-operative breast cancer patients often experience symptoms such as fatigue and anxiety, which hinder their participation in rehabilitation exercises. Social support based on family, peers, and professionals can enhance the compliance of breast cancer patients. Evidence 26 indicates that healthcare providers should strengthen the safety education on exercise for patients and their families, and provide professional health consultation and guidance (20). However, Liu et al. (53) pointed out that most nurses only know the definition, benefits, and progressive principles of exercise, 50% do not know the guidelines for physical activity levels of cancer survivors, 42% are not familiar with the classification of physical activity intensity, and 41% do not know the assessment tools related to physical activity. Therefore, medical institutions should provide support for healthcare providers' mastery of exercise-related knowledge through online seminars, on-the-job training, multidisciplinary education, and scientific research learning. Evidence 27 recommends using digital health technology as an auxiliary means for supervision during the rehabilitation process. Digital behavior change interventions (Digital behavior change interventions, DBCIs) utilize mobile applications, wearable devices, and online platforms to provide technical support for ensuring the rehabilitation outcome through personalized and easily accessible methods that are not limited by time and place (39). Therefore, nurses can consider combining information technology when implementing interventions to provide diversified rehabilitation exercise pathways for post-operative breast cancer patients, such as conducting online synchronous exercise guidance and developing supervision applications combined with wearable technology.

## 5 Limitations

This study synthesizes evidence on Integrated Traditional Chinese and Western Medicine rehabilitation exercises for postoperative breast cancer patients, yet several limitations exist. First, the geographical representativeness of the included literature may be limited, as a considerable proportion of studies originated from China. This may affect the generalizability of the findings to other cultural and healthcare settings. Second, the restriction to Chinese and English literature could introduce language bias and omit relevant evidence. Third，a major concern is the limited number and quality of clinical practice guidelines for the integration of TCM and Western medicine，which restricts the comprehensiveness and robustness of evidence summary. Specifically, there is a lack of high-quality data research on the integration of TCM and Western medicine, the objectivity of the main observation indicators is poor, the observation period is short, and the results lack persuasiveness; the reproducibility of the research results of the integration of TCM and Western medicine is poor, there is a lack of recognized standardized Chinese medical classification standards and quantitative evaluation standards; strict prospective randomized controlled clinical studies are rare, and their quality is generally low. All of these make it difficult to propose high-quality and recommendable opinions on the specific combination methods of TCM and Western medicine when writing the guidelines, and can only rely on low-quality evidence or expert consensus, resulting in weak recommendation strength, reduced external generalizability, and increasing uncertainty in clinical practice. Future research should prioritize well-designed randomized controlled trials and other high-quality studies that evaluate integrated TCM and Western medicine approaches, with particular attention to their potential to improve patients' physical and psychological outcomes, thereby further validating the effectiveness and applicability of the proposed strategies.

## 6 Conclusion

This study summarizes the evidence of integrated traditional Chinese and Western medicine rehabilitation exercises for postoperative breast cancer patients, covering seven aspects: comprehensive assessment, postoperative functional exercise, manual lymphatic drainage, exercise prescription, precautions for special situations during exercise, appropriate traditional Chinese medical techniques, medical supervision and incentive strategies during exercise. It provides evidence-based guidance for healthcare professionals to carry out integrated traditional Chinese and Western medicine rehabilitation exercises. In the future, large-sample, multi-center randomized controlled trials can be conducted to verify the effectiveness of the existing evidence and conduct long-term follow-up on the exercise intervention effects, providing more sufficient basis for the implementation of rehabilitation exercises for breast cancer patients.

## Data Availability

The original contributions presented in the study are included in the article/Supplementary Material, further inquiries can be directed to the corresponding author.
