# Supplementary material for: Integrated TCM and Western medicine rehabilitation program after breast cancer surgery: a best evidence summary
Source: Front Med (Lausanne). 2026 Sep 11;13:1930722. doi: 10.3389/fmed.2026.1930722 (PMC13612453; doi:10.3389/fmed.2026.1930722)
Supplement: Supplementary file 1 [file Table1.docx]

| CNKI | Search strategy | （SU= “乳腺癌”+“乳癌”+“乳房癌”+“乳房肿瘤”+“乳腺肿瘤”）AND（SU=“锻炼”+“康复”+ “运动”+“训练”+ “中医护理”+“中西医结合”+“辨证施护”）AND（SU=“指南”+“共识”+“证据总结”+“系统评价”+“Meta分析”+“随机对照试验”） |
| --- | --- | --- |
|  | result | 252 |
| Pubmed | Search strategy | ("Breast Neoplasms"[MeSH Terms] OR ("breast neoplasm*"[Title/Abstract] OR "breast tumor*"[Title/Abstract] OR "mammary cancer*"[Title/Abstract] OR "breast malignant neoplasm*"[Title/Abstract] OR "breast malignant tumor*"[Title/Abstract] OR "breast carcinoma*"[Title/Abstract] OR "breast ductal carcinoma"[Title/Abstract] OR "breast cancer"[Title/Abstract])) AND ("Exercise"[MeSH Terms] OR ("exercise*"[Title/Abstract] OR "physical exercise*"[Title/Abstract] OR "exercise training*"[Title/Abstract] OR "physical activity*"[Title/Abstract]) OR ("Exercise Therapy"[MeSH Terms] OR ("exercise therapy*"[Title/Abstract] OR "rehabilitation exercise*"[Title/Abstract])) OR ("Resistance Training"[MeSH Terms] OR "strength training"[Title/Abstract]) OR "Integrative Medicine"[MeSH Terms] OR "tcm nursing"[Title/Abstract] OR "traditional chinese medicine nursing"[Title/Abstract]) AND ("Guideline"[Publication Type] OR "practice guideline"[Publication Type] OR "Guideline"[Title/Abstract] OR "practice guideline"[Title/Abstract] OR "clinical practice guideline"[Title/Abstract] OR "Recommendation"[Title/Abstract] OR "Statement"[Title/Abstract] OR "Practice"[Title/Abstract] OR "Standard"[Title/Abstract] OR ("Consensus"[MeSH Terms] OR "Consensus"[Title/Abstract] OR "consensus development"[Title/Abstract]) OR ("systematic review"[Publication Type] OR "systematic review"[Title/Abstract] OR "meta analysis"[Title/Abstract] OR "umbrella review"[Title/Abstract]) OR ("evidence summary"[Title/Abstract] OR "best evidence"[Title/Abstract]) OR ("randomized controlled trial"[Publication Type] OR "randomized controlled trial"[Title/Abstract] OR "RCT"[Title/Abstract] OR "clinical trial"[Title/Abstract])) |
|  | result | 2823个 |
| Wanfang | Search strategy | 检索表达式（中英文扩展&主题词扩展）： 主题:(乳腺癌 OR 乳癌 OR 乳房癌 OR 乳房肿瘤 OR 乳腺肿瘤) and 主题:(锻炼 OR 康复 OR 运动 OR 训练 OR 中医护理 OR 中西医结合 OR 辨证施护) and 主题:(指南 OR 共识 OR 证据总结 OR 系统评价 OR Meta分析 OR 随机对照试验) |
|  | result | 1571 |
| CBM | Search strategy | （"乳腺癌"[常用字段:智能] OR "乳癌"[常用字段:智能] OR "乳房癌"[常用字段:智能] OR "乳房肿瘤"[常用字段:智能] OR "乳腺肿瘤"[常用字段:智能]）AND（"锻炼"[常用字段:智能] OR "康复"[常用字段:智能] OR "运动"[常用字段:智能] OR "训练"[常用字段:智能] OR "中医护理"[常用字段:智能] OR "中西医结合"[常用字段:智能] OR "辨证施护"[常用字段:智能]）AND（"指南"[常用字段:智能] OR "共识"[常用字段:智能] OR "证据总结"[常用字段:智能] OR "系统评价"[常用字段:智能] OR "Meta分析"[常用字段:智能] OR "随机对照试验"[常用字段:智能]） |
|  | result | 459个 |
| VIP | Search strategy | [((((((题名或关键词=乳腺癌 OR 题名或关键词=乳癌) OR 题名或关键词=乳房癌) OR 题名或关键词=乳房肿瘤) OR 题名或关键词=乳腺肿瘤) AND ((((((题名或关键词=锻炼 OR 题名或关键词=康复) OR 题名或关键词=运动) OR 题名或关键词=训练) OR 题名或关键词=中医护理) OR 题名或关键词=中西医结合) OR 题名或关键词=辨证施护)) AND (((((题名或关键词=指南 OR 题名或关键词=共识) OR 题名或关键词=证据总结) OR 题名或关键词=系统评价) OR 题名或关键词=Meta分析) OR 题名或关键词=随机对照试验))](http://202.207.193.112:8000/vpn/2/http/PFVXXZLPF3SYC7UJPAYGG55N/Qikan/search/index?LngMySearHistoryIdGuid=db44a11c-19f7-47f3-8f83-89981ab46b68&from=Qikan_Article_History) |
|  | result | 91 |
| embase | Search strategy | ('breast tumor'/exp OR 'breast tumor' OR 'breast neoplasm*':ab,ti OR 'breast tumor*':ab,ti OR 'mammary cancer*':ab,ti OR 'breast malignant neoplasm*':ab,ti OR 'breast malignant tumor*':ab,ti OR 'breast carcinoma*':ab,ti OR 'breast ductal carcinoma':ab,ti OR 'breast cancer':ab,ti) AND ('practice guideline'/exp OR 'practice guideline' OR 'consensus'/exp OR 'systematic review'/exp OR 'randomized controlled trial'/exp OR 'evidence summary':ab,ti OR 'best evidence':ab,ti) AND ('kinesiotherapy'/exp OR 'kinesiotherapy' OR 'integrative medicine'/exp OR 'integrative medicine' OR 'tcm nursing':ab,ti OR 'traditional chinese medicine nursing':ab,ti) |
|  | result | 1553个 |
| Cochrane library  Cochrane library | Search strategy | #1 MeSH descriptor: [Breast Neoplasms] explode all trees 21300  #2 (breast tumor*):ti,ab,kw OR (mammary cancer*):ti,ab,kw OR (breast malignant neoplasm*):ti,ab,kw OR (breast malignant tumor*):ti,ab,kw OR (breast carcinoma*):ti,ab,kw OR (breast ductal carcinoma):ti,ab,kw OR (breast cancer):ti,ab,kw 50991  #3 #1 OR #2 51889  #4 MeSH descriptor: [Exercise] explode all trees 41560  #5 MeSH descriptor: [Exercise Therapy] explode all trees 23860  #6 MeSH descriptor: [Integrative Medicine] explode all trees 124  #7 (Integrative Medicine):ti,ab,kw OR (tcm nursing):ti,ab,kw OR (traditional chinese medicine nursing):ti,ab,kw 2345  #8 #4 OR #5 OR #6 OR #7 55279  #9 #3 AND #8 1230 |
|  | result | 1211 |
| JBI | Search strategy |  |
|  | result | 10 |
| CINAHL | Search strategy | S1（130218）:[SU ("Breast Neoplasms") OR TI ("breast neoplasm" OR "breast tumor" OR "mammary cancer" OR "breast malignant neoplasm" OR "breast malignant tumor" OR "breast carcinoma" OR "breast ductal carcinoma" OR "breast cancer") OR AB ("breast neoplasm" OR "breast tumor" OR "mammary cancer" OR "breast malignant neoplasm" OR "breast malignant tumor" OR "breast carcinoma" OR "breast ductal carcinoma" OR "breast cancer")](https://research.ebsco.com/search/results?db=ccm&expanders=concept&limiters=None&searchMode=boolean&sort=relevance&sqId=sq:5381fde3-81bc-4e3e-a1db-b4434b878a7e&userDirectAction=true)  S2（269204）:SU ("Exercise") OR TI ("exercise" OR "physical exercise" OR "exercise training" OR "physical activity") OR AB ("exercise" OR "physical exercise" OR "exercise training" OR "physical activity")  S3（5804）:[SU ("Integrative Medicine") OR TI ("tcm nursing" OR "traditional chinese medicine nursing") OR AB ("tcm nursing" OR "traditional chinese medicine nursing")](https://research.ebsco.com/search/results?db=ccm&expanders=concept&limiters=None&searchMode=boolean&sort=relevance&sqId=sq:5e04ad3a-b816-4b69-b4a8-9fda417b1500&userDirectAction=true)  S4（274769）:[S2 OR S3](https://research.ebsco.com/search/results?combinedSearchQueryId=sq:ecc8a337-9d68-45f3-bddb-5c44192818d4&db=ccm&expanders=concept&limiters=None&searchMode=boolean&sort=relevance&sqId=sq:ecc8a337-9d68-45f3-bddb-5c44192818d4&userDirectAction=true)  S5（4280）:[S1 AND S4](https://research.ebsco.com/search/results?combinedSearchQueryId=sq:b67f8e74-de2a-4362-9310-17b1f8d0baf1&db=ccm&expanders=concept&limiters=None&searchMode=boolean&sort=relevance&sqId=sq:b67f8e74-de2a-4362-9310-17b1f8d0baf1&userDirectAction=true)  S6（183591）:[SU ("Guideline") OR SU ("Recommendation") OR SU ("Consensus") OR SU ("systematic review") OR SU ("evidence summary")](https://research.ebsco.com/search/results?db=ccm&expanders=concept&limiters=None&searchMode=boolean&sort=relevance&sqId=sq:e3e62c84-b60b-4075-8955-0398bec94b51&userDirectAction=true)  S7（275）:[S5 AND S6](https://research.ebsco.com/search/results?combinedSearchQueryId=sq:40e99ca6-ee63-4304-b186-9c0e6a823b2c&db=ccm&expanders=concept&limiters=None&searchMode=boolean&sort=relevance&sqId=sq:40e99ca6-ee63-4304-b186-9c0e6a823b2c&userDirectAction=true) |
|  | result | 275 |
